# The Association between Bilateral Deficit and Athletic Performance: A Brief Review

**DOI:** 10.3390/sports10080112

**Published:** 2022-07-27

**Authors:** Petra Železnik, Vita Slak, Žiga Kozinc, Nejc Šarabon

**Affiliations:** 1 Faculty of Health Sciences, University of Primorska, SI-6310 Izola, Slovenia; 97210503@student.upr.si (P.Ž.); 97210477@student.upr.si (V.S.); nejc.sarabon@fvz.upr.si (N.Š.); 2 Andrej Marušič Institute, University of Primorska, Muzejski trg 2, SI-6000 Koper, Slovenia; 3 Human Health Department, InnoRenew CoE, Livade 6, SI-6310 Izola, Slovenia; 4 Laboratory for Motor Control and Motor Behavior, S2P, Science to Practice, Ltd., Tehnološki Park 19, SI-1000 Ljubljana, Slovenia

**Keywords:** bilateral index, bilateral facilitation, athletic performance, muscle performance

## Abstract

An abundance of information can be found in the scientific literature regarding the bilateral deficit (BLD) in different contraction types, including its possible underlying mechanisms. On the other hand, studies on the relationship between BLD and athletic performance have only begun to emerge in recent years. The purpose of this review article is to assemble and analyze the literature on the topic of the relationship between BLD and athletic performance. After a detailed review of the scientific databases, we analyzed 10 relevant scientific articles. BLD calculated from outcomes of vertical jumps was positively related to the ability to change direction quickly in volleyball, basketball, tennis and student population, but not in soccer. Sprint running performance does not seem to be associated with BLD, while one study suggested that a smaller BLD is associated with a more efficient start in a sprint. Apart from the associations with change in direction performance, there is little evidence to support the association between BLD and athletic performance; thus, further research is required in other sports, incorporating sport-specific performance outcomes and multiple tasks to calculate the BLD.

## 1. Introduction

Humans can perform certain movements unilaterally or bilaterally. Henry and Smith [1] were the first to establish that the force production during a simultaneous maximal voluntary contraction of both limbs tends to be lower than the sum of the forces produced by the left and right limbs separately (unilateral contraction). Research on the bilateral deficit (BLD) is possible when comparable unilateral and bilateral tasks are performed, such as unilateral and bilateral squat jumps (SJ) [2] or unilateral and bilateral measurements of muscle strength [3]. In addition to BLD being present during maximal voluntary contraction, studies have suggested that a similar deficit in the bilateral execution of movement tasks also occurs in terms of reaction times [4]. The BLD is usually determined by the value of the bilateral index (BI) obtained by the following equation:

$$\mathrm{BI}\;\left[\%\right]=\left(100\times \frac{\mathrm{bilateral}}{\mathrm{right}\;\mathrm{unilateral}\;+\;\mathrm{left}\;\mathrm{unilateral}}\right)-100,$$

where bilateral means the sum of the forces produced by each limb during bilateral contraction, while unilateral means the sums of maximum forces produced during unilateral contractions with each limb [2,5]. Negative BI values indicate the presence of BLD, while positive values indicate the presence of a reverse phenomenon, i.e., bilateral facilitation [6]. Bilateral facilitation thus represents a situation in which the maximal voluntary force, produced in bilateral conditions, is greater than the sum of the forces, produced in unilateral conditions [2,5].

The presence of BLD has been proven in various types of muscle contractions, both isometric and slow and explosive dynamic contractions [7]. The occurrence of BLD in dynamic single-joint movements has been reported consistently regardless of whether the contraction is eccentric or concentric [5,8]. The magnitude of BLD during concentric or eccentric contractions is on average around 10%, with the magnitude increasing with enhancing the speed of movement [5]. BLD is more inconsistent in isometric conditions compared to dynamic ones, with the most discrepancies existing among studies occurring in knee extensors [2,5]. The occurrence of BLD was also confirmed in ballistic contractions, that is, in SJ, countermovement jump (CMJ), horizontal countermovement jump, and drop jump (DJ) [2,5,7,8]. When jumping, BLD may refer to the difference in the height of a two-leg jump compared to the sum of the heights of one-legged jumps, or it may be derived from other jump metrics (e.g., mean or peak power, mean or peak force, phase-specific force impulse variables, or rate of force development) [7]. Many possible mechanisms are proposed to explain the phenomenon of BLD, although most likely several factors contribute to its occurrence. Škarabot et al. [5] divided the possibly underlying factors into four categories: psychological factors, factors related to the measurement task, physiological factors, and neurophysiological factors. The detailed mechanics of the BLD are yet to be determined, but to date, the most widely accepted explanation of deficit is interhemispheric inhibition—a phenomenon that describes the inhibition of opposing cerebral hemispheres [5,6,9,10].

The phenomenon of bilateral facilitation suggests that BLD is a highly plastic quality. Several studies have shown that BLD can be altered by resistance training. More specifically, unilateral exercises increase the magnitude of BLD, while bilateral exercises decrease it [11,12,13]. Based on these findings, it could be speculated that BLD could be associated with physical and motor performance [5]. This may apply to specific sports, where a lower BLD value may be more favorable in sports in which individuals perform mostly bilateral movements (e.g., rowing, weightlifting, and ski jumping), while a higher BLD value could be beneficial in sports that require mostly unilateral movement (e.g., changes of direction, throws, and high or long jumps) [2,8].

Research on the underlying mechanisms of BLD has abounded, and many studies have addressed the impact of different forms of resistance training on BLD. However, one research area that has been somewhat neglected involves performance, with the question being can BLD influence athletic performance? In a scoping review from 2016, Škarabot et al. [5] identified only one study that investigated the influence of BLD on athletic performance, and stressed that this should be addressed in the future. This question is extremely relevant for coaches and other associates in sports practice, for if a connection between BLD and athletic performance is established, BLD could be used as one of the indicators for designing and monitoring training performance. In the recent years, a high number of studies on this topic has finally emerged; thus, a new review synthesizing the evidence collected to date is needed to help to transfer the knowledge to practitioners and identify remaining gaps in the literature and promising research directions. The purpose of this article is thus to review of the literature on the topic of the relationship between BLD and athletic performance.

## 2. Materials and Methods

To obtain an answer to the study question, we aimed to collect all the studies that investigated the relationship between BLD and athletic performance. Because of the high heterogeneity of the existing studies regarding performance outcomes, approaches to obtain BLD outcomes and included populations, we did not perform a classic systematic review with meta-analysis; rather, we collected and qualitatively analyzed the relevant studies. We searched PubMed and Google Scholar databases in February 2022 using the following keywords: *bilateral deficit AND athletic performance.* We included articles that were available in full-text and were written in English. The inclusion and exclusion criteria were structured using a PICOS tool [14], as follows:

- Population: recreational and elite athletes. No exclusion regarding sex, ethnicity and level of play.
- Intervention: N/A; the research question requires cross-sectional evidence.
- Comparison: N/A; the research question requires cross-sectional evidence.
- Outcomes: The studies had to report at least one BLD outcomes (calculated from isometric, isokinetic or isotonic tasks assessed with dynamometers, or ballistics tasks, such as jumps) and at least one outcome measure that describes sport-specific performance or can be considered as a proxy of general performance in sport (e.g., CoD tasks, sprints, and jumps).
- Study design: cross-sectional correlational studies.

The relevance of the search results was first checked by title, then by reading the abstracts. If they were appropriate, the articles were read in full and, if relevant, were included in the final analysis. Additionally, the reference lists of previous reviews on the topic [5,9] and the reference lists of the already included articles were examined to identify any potential studies that might fit the criteria. All stages of the search were performed by two authors (PŽ and VS) and potential disagreements were resolved by consulting the other two authors. From every study, we extracted: (a) participant characteristics (age, sex, anthropometrics, athletic discipline, and level of play); (b) descriptors of testing procedure (tests that were used to calculate BLD and tests that were used as proxies for athletic performance) and (c) correlation coefficients between BLD outcomes and performance outcomes.

## 3. Results

The database search yielded 105 relevant articles. After reviewing the titles, 32 articles were relevant, after reviewing the abstracts, 19, and after reviewing the entire articles, 7 were relevant. Additionally, the reference lists of relevant articles were examined and one other suitable article fit the criteria. Therefore, eight scientific articles were included in the final review. The authors were in 100% agreement regarding the papers included in the final stage of the review. The results of the included articles are shown in Table 1.

## 4. Discussion

The aim of this paper was to review the literature on the relationship between BLD and athletic performance. We established that the effect of BLD depends on the type of athletic performance being evaluated. Smaller BLD is beneficial for athletes that perform predominantly bilateral actions, while in most team sports (where the majority of movement patterns are performed unilaterally), a larger BLD is likely to be advantageous. However, studies are somewhat inconsistent and report mostly small to moderate associations between BLD and athletic performance tests.

Recent studies about BLD concerning athletic performance focus primarily on sprint velocity and CoD. Ascenzi et al. [8] investigated the correlations between BLD in SJ and horizontal CMJ with sprint velocity and CoD in professional soccer players and reported no statistically significant correlations. The lack of correlations between BLD and sprint performance was also reported by Bishop et al. [2] who used a variety of jumps to calculate BLD. Nicholson and Masini [18] further report the absence of links between BLD in SJ and CoD. On the other hand, a study on elite runners found a connection between BLD and sprint velocity (Bračič, Supej, Peharec, Bačić, and Čoh, 2010). The methods included a measure of the production of force on the starting block at the start of the sprint, based on which the authors report that subjects with a lower BLD in CMJ produced a greater force impulse on the starting blocks. Smaller BLD in maximum power output in SJ has also been shown to be beneficial in performing bilateral CMJ (both in terms of maximum force as well as maximum and average power) [18]. In contrast to the aforementioned findings, three studies reported a moderate association between BLD in CMJ and CoD [2,17,19], indicating that higher BLD is beneficial for CoD performance. Contradictory results may be due to diverse movement tasks to evaluate BLD and athletic performance. The type of task affects the speed and direction of movement, and thus various factors of BLD [8].

Another factor that could explain the discrepancy between the studies is that diverse populations were included. While we can suggest that a larger BLD could be beneficial for the performance of unilateral actions, such as changes of direction or one-legged jumps, we need to be somewhat reluctant in the latter finding, as some studies have not confirmed this association. Specifically, the studies that confirmed the positive influence of BLD on CoD in volleyball players [19], basketball and tennis players [17] and university students [2], but not soccer players [8]. These results are difficult to explain, as basketball, tennis and soccer are all characterized by high prevalence of CoD actions [20,21]. Nevertheless, further research on association between BLD and CoD is needed in all sports. For now, we can recommend to the practitioners to emphasize unilateral strength for improving CoD in basketball and tennis, but not soccer, while the association between BLD and CoD is currently unknown for other sports.

The potential benefits of BLD in team sports are also corroborated in the training study of Gonzalo-Skok et al. [13] conducted on young basketball players. The study compared training sessions, including either predominantly unilateral or predominantly bilateral exercises on athletic performance, while monitoring BLD. As expected, in a group that trained unilaterally, BLD augmented. Jump performance, sprint velocity and maximal power amplified in both training groups. Unilateral training promoted adjustments that improved jumps, linear sprint and CoD speed, which are crucial elements in basketball and other team sports [13]. We can conclude that the relation of BLD with athletic performance depends on the requirements of the sport. If unilateral performances are significant for enhancing athletic performance, it may be better to increase BLD with unilateral exercises [7,17]. The exact mechanisms underlying the associations between BLD and unilateral performance are difficult to determine. Neurophysiological factors, such as inter-hemispheric inhibition, antagonist coactivation and spinal excitability, could play a role in this association [5]. From the neurological perspective, the BLD has been sometimes viewed as “unilateral facilitation” [5], which can be increased with unilateral training [11,13]. In addition, when BLD is derived from dynamic tasks, such as jumps, neuromechanical factors, such as the force–velocity relationship, could play a role. Namely, the BLD in jumping is largely explained by force and velocity capacities of the individual [22], while the force–velocity relationship has been shown to be related to sports performance many times [23,24].

There are only a few studies that included specifically designed performance tests for a single sport. Turnes et al. [3] reported no connections between BLD in hand grip strength and sport-specific test designed for judoists. On the other hand, Kons et al. [16] reported poorer performance in a special judo test in subjects with higher BLD in CMJ. In the aforementioned sport-specific test, the subject must perform as many throws (ippons) of the opponent as possible in a limited time. Given that ippons consists of continuous movements that requires coordinated force production from upper and lower extremities, this finding seems reasonable. Other tests in the study were not associated with BLD. The study suggests that it might be intelligible to reduce BLD in CMJ in judo. In sum, the BLD could negatively affect judo performance, while none of the studies reported any positive influences. Minimizing BLD in CMJ for judoists can be recommended. An area of research that needs to expand in the future is the relationship between BLD and sport-specific tests, as current studies mostly focus on CoD, sprint and jumping tasks only.

In addition to sports activities, BLD also affects the functional ability to perform daily bilateral tasks. In a study on older adults, Ruiz-Cardenas et al. [25] investigated the association of BLD with the success of performing the sit-to-stand test in menopausal women. The outcomes show that BLD in the rate of force development in the first 50 ms was associated with a poorer performance of sit-to-stand test; thus, it is coherent to maintain explosiveness in older adults, therefore empowering them to be independent in daily tasks. While this study was not included in our review as it included a non-athletic population, it shows the potential association between BLD derived from rate of force development and neuromuscular performance; therefore, the rate of force development should also be considered in future studies assessing the influence of BLD on athletic performance.

In accordance with the previous literature, several studies included in our systematic review confirmed the large impact of movement task and output variables on BLD and its connections to athletic performance [2,3,8,18], with BLD most commonly measured in various types of jumps. The measurements in included articles seldom incorporated the measures of muscle strength via the maximal voluntary isometric contraction. BLD in this type of task does not appear to be related to the athletic performance of athletes [3,18]. Considering BLD as a measure of athletic performance, it would be reasonable to perform measurements in several planes and during sports-specific movements. In addition to the diversity of motor tasks, the variables included in the BLD calculation also varied. The methods used in the studies to calculate BLD include a measure of height or length of the jump [2,17], maximum power or force [3,18,25,26], reaction time [4], and the rate of force or torque development [25,26]. In the future, the most suitable variables for BLD calculation should be determined. Kozinc and Šarabon [17] reported that the relation between the CoD velocity and BLD was greater when BLD was calculated based on the force impulse at CMJ, compared to jump height and maximum power.

Current research on BLD was mostly conducted on subjects of a variety of physical fitness levels (elite or recreational athletes), diverse age groups (from younger athletes to menopausal women), sample sizes (10 to 165 subjects), and a variety of movement tasks with which the athletic performance was evaluated (sprint velocity, CoD, horizontal and vertical jumps, sit-to-stand, and reaction time test). Hence, while interpreting the results, we must consider these differences. In the future, the studies should therefore be conducted on larger samples, on the one hand in athletes with predominantly bilateral, and on the other, predominantly unilateral elements, and to compare the impact of their BLD on sport-specific performance. Moreover, future research should consider incorporating multiple tasks to assess BLD. In particular, isometric and isokinetic tasks were scarcely employed and most of the included studies used jumping tasks to obtain BLD.

## 5. Conclusions

The relationship between BLD and athletic performance is not yet fully understood. Current studies mainly include sprint and ability to quickly change direction as performance measurements, in which the results indicate the positive effect of larger BLD in tennis, basketball and volleyball (the results for soccer are inconclusive). On the other hand, BLD seems to be undesirable in judo. In sports, where predominantly unilateral actions are performed, larger BLD might be beneficial to augment athletic performance as elements of acceleration, braking, jumping, and changing direction are essential elements for successful participation in most team sports. If the requirements of sport for unilateral activities are high, it may be appropriate to increase the BLD with unilateral exercises. There is a considerable amount of literature on BLD, but only a small proportion of studies focus on the connection between BLD and athletic performance. Future research could therefore be conducted on athletes of different disciplines with standardized measurement protocols, which would provide transparent answers about the impact of BLD on athletic performance. Furthermore, it would be convenient to examine whether BLD can be used as a guide in training design, for example, in terms of the proportion of unilateral and bilateral exercises involved. With an increasing number of studies, we will eventually be able to perform a systematic review with meta-analysis, with possible subgroup analyses that will answer the remaining questions.

## Figures and Tables

**Table 1:** Summary of the study findings. Statistically significant (at *p* < 0.05) correlations are in bold text.

Reference	Subjects	Outcomes for BLD Calculation	Performance Indicators	Findings
Ascenzi et al., 2020 [8]	27 young male soccer players (age: 18.5 ± 0.6 years)	Peak and average power in SJ with various loads and CMJ	90° CoD (10 + 10 m) and CoD deficit; sprint times (10, 20, 30, 40 m)	-No statistical relationships between BLD and speed assessment (COD or sprint), except a moderate correlation between CoD time and BLD in SJ on the right leg with additional 25% body weight (r = **−0.45**).
Bishop et al., 2021 [2]	18 recreationally trained, male university students (age: 25.5 ± 3.8 years)	CMJ and DJ height, peak power, peak force, eccentric and concentric impulse	505 CoD test, Cod deficit, 10 and 30 m sprint	-A higher BLD in CMJ jump height and concentric impulse is related to faster 505 test (r-left = **−0.48** and **−0.51**; r-right = **−0.53** and **−0.64**), and CoD deficit (only right leg, **−0.59** and **−0.60**), but not linear speed at 10 m (|r| = 0.09–0.38) and 30 m (|r| = 0.15–0.31). -BLD in DJ flight time related to better 505 test time (r = **−0.48**). -BLD in DJ not related CoD deficit (|r| = 0.03–0.39) nor linear sprinting (|r| = 0.09–0.43).
Bračič et al., 2010 [15]	12 male elite sprinters (age: 22.49 ± 3.39 years)	CMJ height, take-off velocity and peak force	Peak force and total in the double start of the sprint start	-Lower values of BLD were associated with a higher peak force production of the rear leg in sprint start (r = **−0.63**) and higher total impulse of force on the blocks (r = **−0.55**).
Kons et al., 2022 [16]	14 male judo athletes (age: 25.2 ± 2.2 years)	CMJ height, peak power, average power, peak force, peak velocity, impulse	Special Judo Fitness Test (total throws the with ippon-seoi-nage technique in 15 + 30 + 30 s)	-Negative correlations between specific judo test of opponent’s total throws and BLD in CMJ jump height (r = **−0.61**), peak force (r = **−0.69**), mean power (r = **−0.85**), peak power (r = **−0.53**), peak velocity (r = **−0.73**) and impulse (r = **−0.61**). -No correlation between BLD and tests of speed and maximal aerobic speed (all |r| < 0.40).
Kozinc and Šarabon 2021 [17]	165 young basketball players (105 male, age: 16.7 ± 1.2 years; 60 female, age: 17.9 ± 13.7 years) and 95 young tennis players (56 male, age: 16.8 ± 1.6 years; 39 female, age:15.7 ± 3.1 years)	CMJ height, peak power and phase-specific force impulse metric	505 CoD test	-Several statistically significant relationships were found between 505 CoD performance and BLD for male athletes (r = **0.21–0.52**).
Nicholson and Masini 2021 [18]	10 physically active males (age: 23 ± 1.3 years)	Isometric leg extension peak force and rate of force development, SJ height, peak force, peak power and average power	CMJ height, peak force, peak power and average power, 135° change of direction test (10 m approach + 5 m)	-Lower BLD in SJ peak power is associated with a greater peak force (r = **0.73**) and peak power (r = **0.75**) in CMJ. -BLD in isometric leg extension force is not related to CMJ height, power and force (|r| = 0.14–0.46) nor to CoD outcomes (|r| = 0.06–0.32).
Pleša et al., 2022 [19]	47 young volleyball players; age: 20.8 ± 3.8 years	CMJ height, peak power and phase-specific force impulse metric.	Modified *t*-test, 505 CoD test, 25 m sprints (with 5, 10, and 15 splits), and vertical approach jumps	-Small to moderate correlations between CMJ BLD outcomes and 505 CoD test (r = **0.36–0.47**). -BLD in CMJ peak power related to smaller CoD deficit (r = **−0.42**). -BLD in CMJ height related superior sprint on 10 m (r = **0.33**), 15 m (r = **0.36**) and 25 m (r = **0.49**), but not 5 m (r = 0.18). -Modified *t*-test performance not correlated with BLD variables (r = −0.15–0.22).
Turnes et al., 2019 [3]	19 male judo athletes (age: 22.1 ± 4.6 years)	Isometric handgrip strength test	Special Judo Fitness Test (total throws the with ippon-seoi-nage technique in 15 + 30 + 30 s)	-Correlations between performance at judo tests and BLD in handgrip strength tests were not significant (r ≤ 0.27).

BLD—bilateral deficit; SJ—squat jump; CoD—change of direction; CMJ—countermovement jump; DJ—drop jump.

## Data Availability

Not applicable.
